# Enhanced Primary Health Care Intervention: Perceived Sustainability and Challenges Among Implementers

**DOI:** 10.1177/21501327211014096

**Published:** 2021-05-08

**Authors:** Komathi Perialathan, Mohammad Zabri Johari, Norrafizah Jaafar, Kong Yuke Lin, Low Lee Lan, Nur Aliyah Sodri, Siti Nur Nabilah Mohd Yunus

**Affiliations:** 1 Ministry of Health Malaysia, Setia Alam, Selangor, Malaysia

**Keywords:** sustainability, primary health care, health care provider, EnPHC

## Abstract

**Purpose:**

This study aimed to assess and explore perceived sustainability and challenges of the intervention among Health Care Providers (HCPs) who were involved.

**Methods:**

The study applied mixed-method embedded design to analyze both quantitative and qualitative data. Quantitative approach was used to evaluate sustainability perception from 20 intervention clinics via self-reported assessment form whereas qualitative data were obtained through in-depth interview (IDI) and focus group discussions (FGDs) 14 health care professionals participated in IDI session and were either care coordinators, liaison officers (LOs)/clinic managers, or medical officers-in-charge for the clinic’s intervention. Nine FGDs conducted comprised 58 HCPs from various categories.

**Results:**

HCPs from all the 20 clinics involved responded to each listed Enhanced Primary Healthcare (EnPHC) intervention components as being implemented but the perceived sustainability of these implementation varies between them. Quantitative feedback showed sustainable interventions included risk stratification, non-communicable disease (NCD) screening form, referral within clinics and hospitals, family health team (FHT), MTAC services and mechanisms and medical adherence status. Qualitative feedback highlighted implementation of each intervention components comes with its challenges, and most of it are related to inadequate resources and facilities in clinic. HCPs made initiatives to adapt based on clinical setting to implement the interventions at best level possible, whereby this seems to be one of the core values for sustainability.

**Conclusion:**

Overall perceptions among HCPs on sustainability of EnPHC interventions are highly influenced by current experiences with existing resources. Components perceived to have inadequate resources are seen as a challenge to sustain. It’s crucial for stakeholders to understand implications affecting implementation process if concerns raised are not addressed and allocation of needed resources to ensure overall successfulness and long term sustainability.

## Background

Non-communicable diseases (NCDs) accounts as the main cause of deaths worldwide; more so than communicable diseases. More than 36 million die annually from NCD related diseases including 14 million premature deaths, and most of these premature deaths occurs in lower and middle-income countries.^1^ In low-income countries, the rapid rise in NCDs is projected to hinder poverty reduction initiatives, especially by rising health care-related household costs. The steep cost of NCDs, including frequent, prolonged and costly care and loss of breadwinners, annually forces millions of people into poverty and represses development.^2^ Malaysia is facing and increasing upwards trend of NCD prevalence. The two most recent disease burden surveys revealed a 5.9% increase of diabetes prevalence from 11.6% in 2006 to 17.5% in 2015 and a 19.5% increase in hypercholesterolemia from 28.2% in 2006 to 47.7% in 2015.^3^ To address these issues, a cost effective and evidence-based interventions are needed to better manage and possibly allay the increasing trend of the NCDs especially at the primary health care setting.

Malaysia’s primary health care scenario is not that far different and is presently suffering from increasing burden from NCD patient; taxing the health service providers and the system itself. Concerns for poor referral mechanism, lack of personalized care, low patient satisfaction and the need for quality primary health care has led to the introduction of a comprehensive integrated and multi-approach intervention package known as the Enhanced Primary Healthcare (EnPHC) in July 2017. This intervention had four key objectives—optimization of comprehensive care, service delivery through structural and operational integration, targets and managements of NCD risk, and multi sectoral interventions for coordinated response. The primary care level intervention focused on establishment of Family Health Teams (FHTs) who are accountable for individuals and population within catchment areas, initiation of multidisciplinary healthcare team, create strategic mechanism for integrated referral and networking of providers, enhancement of technology use and in overall develops the leadership capabilities at health clinics.^4^

Intervention through primary care reform is now seen as a fundamental approach in addressing healthcare issues globally.^5^ Incorporating integrated approaches in the existing health care system such as screening and early detection at community level, diseases management and healthy lifestyle promotion at primary care level and continuous monitoring of patient’s outcome through family health care system are important elements to ensure successfulness of these intervention.^6^ A well-supported primary healthcare is seen as the key aspect for an efficient and effective patient-centered healthcare system.^7^ Evidence shows that reformation of national health care systems through strengthening of primary care infrastructures results in healthier populations, fewer health-related disparities and lower overall costs for health care.^8^ However, in most cases, little is known about the sustainability of these reforms. Sustainability is defined as the extent to which a newly implemented treatment, service, practice, or innovation is maintained or institutionalized within a service^9^ and often, it is a major challenge for policy makers in healthcare to continuously sustain an effective complex intervention.^10^

A recent review showed that up to 60% of new interventions cease when funding ends.^11^ Such phenomenon leads to significant negative outcomes; waste of resources, inability to provide best practices and healthcare providers being cynical about the change which ends in poor trust between the initiators of change and implementer at the ground level.^12^ Perception, attitudes and behaviors of healthcare providers are some key contributing factors for acceptance or refusal of change in any newly introduced healthcare intervention,^1^ which may result in the organization reverting back to its old ways once the implementation period has ended.^13^ Therefore, it is important for change programmers to be able to identify and cope with the mounting stressors to ensure the sustainability of these interventions.^14^ The assessment of challenges and sustainability will enable future uptake of the intervention to be implemented accordingly to the specific set ups of the locality.

This paper specifically focuses on the perceived sustainability of the newly introduced EnPHC intervention at the 20 selected primary care health clinics in Selangor and Jofor. Apart from analyzing their rating on the level of easiness in sustaining each intervention component under EnPHC, implementers shared as well their perceptions on issues that affects or strengthen the sustainability of these interventions.

## Methodology

### Study Design

This study is a subset of a larger process evaluation study and for this study a mixed methods approach with embedded design by integrating both quantitative and qualitative approaches. The quantitative approach assessed the perceived sustainability of implementers at 20 selected primary health care clinics in 2 Malaysian States—Johor and Selangor and focusing in regards to each intervention component under the EnPHC. Participants are healthcare providers (HCPs) who are clinic managers, liaison officers (LOs), clinic care coordinators and clinic staffs involved in the EnPHC implementation. The sustainability assessment was conducted from April to June 2018, 10 months after the initial implementation in July 2017. The time frame assessment selection was to enable implementers to better express changes and projection towards sustainability of the integrated intervention after experiencing it for more than 10 months. The study was registered under National Medical Research Register, Ministry of Health Malaysia (NMRR-17-295-34771), and was approved by Medical Research and Ethics Committee (MREC) Ministry of Health Malaysia.

### Data Collection and Materials

Quantitative data was collected using a pre-tested self-reported assessment form (PE-03) to evaluate implementers perception on sustainability rating of the EnPHC intervention at their clinic. The form was mailed to the appointed LOs or clinic managers at the 20 intervention clinics. The appointed LOs or clinic managers are in charge of the EnPHC interventions at the clinic and were asked to rate all the listed intervention using a 4 Likert Scale point—their perceived sustainability for each intervention; from very easy, easy, challenging, or very challenging to sustain the interventions based on their own experience at their facility.^15^

Qualitative data were obtained through in-depth interview (IDI) and focus group discussion (FGD) among HCPs at 8 selected intervention clinics using purposive sampling method. The selected clinics were randomized and matched for characteristics and outcomes of EnPHC intervention in both States. A semi-structured interview guide for HCPs consisting topic on acceptance, adaptation, feasibility, sustainability, and suggestion to improve the intervention components was used during the session. HCPs perspective in foreseeing the intervention’s capacity to be sustained and implemented in a bigger scale was explored during this session. A total of 14 IDI and 9 FGD sessions (comprising of 5-8 HCPs per session) were conducted and total of 58 HCPs (25 professionals consisting medical doctors and pharmacists; and 33 paramedics consisting of Nurses, Assistant Medical Officer and other clinic staff (clerks, health attendants, lab staff) participated. Participants were recruited until data saturation is reached. Maximum variation sampling was used by varying the clinic settings, and HCPs with different job functions. The IDI and FGD sessions were conducted by research team members who had prior experiences in conducting qualitative based study and who were also not a close acquaintance with any of the participants to avoid potential response bias. The interview sessions were conducted face-to-face and participatory and recording permission were obtained prior to the IDI and FGD sessions. The duration for IDI sessions were from 30 to 120 min whereas for FGD was from 120 to 180 min. To preserve confidentiality, all identifiers to the clinics and study participants were anonymized and participants were given a code.

**Figure 1.**
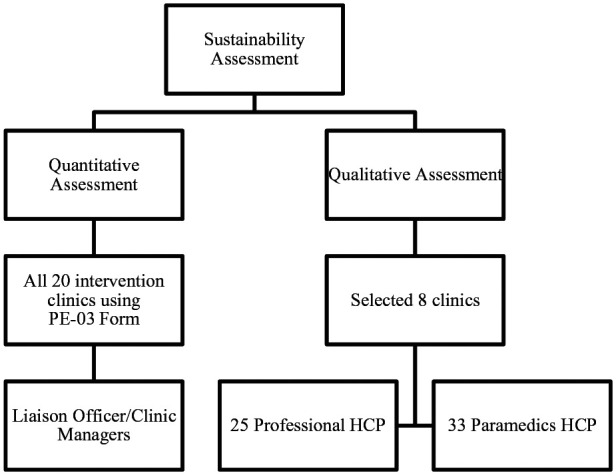
Enrollment process for sustainability assessment.

### Data Analysis

The quantitative data retrieved through the self-reported assessment form were tabulated into Microsoft Excel using simple descriptive analysis. The self-reporting form assessed the perceptions of clinic managers or LOs on how they would rate the sustainability of each intervention components in the long term and the data are presented in the form of percentage. The 4 Likert rating feedback from these forms was compressed to only 2 categories. Responses for 1 (very challenging) and 2 (challenging) was collapsed into one category as “challenging to sustain” and responses for 3 (easy) and 4 (very easy) was collapsed to one category as “easy to sustain.” Percentage represents feedback numbers on perceived sustainability for every intervention component that were listed, whether the intervention easy or challenging to sustain.

Qualitative data management was facilitated through NVivo11™. To ensure reliability of qualitative data, all audio transcribed data were read several times along with the interview audio-recording file by independent transcriber and researchers to ensure accuracy of transcripts. Verbatim transcripts were also cross-checked with field notes taken during the data collection process. Data was analyzed thematically and coded based on the issues presented after consensus was made among the researchers based on their research lens and field of expertise.^15^

## Result

The main scopes of the intervention components assessed for the quantitative assessment consisted of triaging system, introduction to care coordinator roles, NCD screening and NCD risk stratification, referral system, family health, and pharmacy team. In qualitative exploration, HCPs from various categories at the clinics; professionals, assistant medical officers (MA), staff nurses, pharmacists, and other clinic staff for example, clerk, attendant, lab paramedics shared issues affecting the sustainability of each intervention component and how in some cases, adaptations were made to implement the intervention. HCPs shared as well their reflections on the current workflow and prior to the intervention.

**Figure 2.**
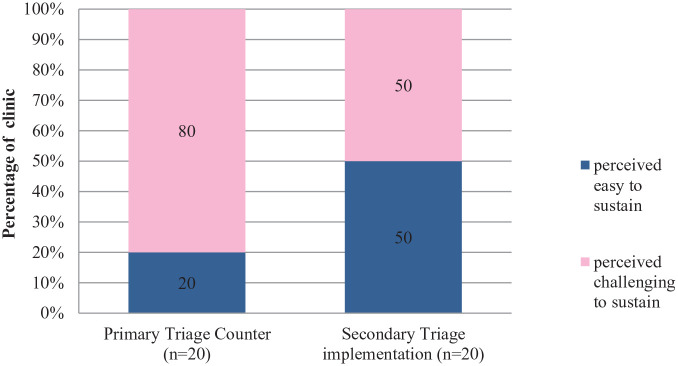
Perceived sustainability of triaging system.

Under EnPHC, two-tiered triaging system was introduced; Primary triage and Secondary Triage with its own roles and functionality. Primary triage in clinic functions mainly in channelling arriving patients according to their service needs. Staff nurses or assistant medical officers (MA) are recommended to manage the primary triage counter, as their medical knowledge and skill will assist in identifying patients need and treated accordingly by the severity of their health conditions. At the secondary triage counter, most of the EnPHC interventions components are constituted here such as early NCD screening, NCD risk stratification activities, health education by paramedics, and establishment of NCD patients’ care continuity.^15^

Based on the quantitative feedback received, all 20 clinics implemented this triaging system and majority (80%) foresee it as challenging to sustain due to limited space availability in the clinics and shortage of staffs. In the qualitative evaluation, clinic managers shared that due to lack of staff, they had difficulties in arranging staffs like Medical Assistants (MA) to handle the primary triage because during the peak hours of clinic, the medical assistants are more needed in the clinical service. However, to ensure the continuation the proposed intervention, some clinics managed and adjusted based on their clinic setting by arranging schedules or replace with other staffs. The clinic managers also felt that primary triage facilitated the process of screening patients according to the types of cases.

*“*. . .*so what I can see is that primary triage, the use of it is to reduce waiting time, we can screen patient. . . hmmm normal waiting time, emergency waiting time and so on. It’s just that everyday I still see patient waiting for so long even if we have primary triage. I’m not sure what’s the problem but I think every partly, it’s due to manpower.” (Professional)*

For Secondary Triage, quantitative assessment shows it was an equal response on both side, half of them (50%) felt it is challenging and half of them (50%) felt it is easy to sustain. In the qualitative feedback, after 10 months of implementation, the main challenge cited in implementing this intervention were similar to primary triage; unavailability of appropriate space in clinic especially old clinics with smaller structure and space, shortage of staff and cause an increase in patients’ waiting time.

“*After EnPHC, the patient waiting time is longer. I have only one PPK (Health Attendants) left. . . patient blood has to be taken twice, HbA1c 4 times. There are many bloods taken but not filed up yet. So, when patient comes, then will start searching for their file, searching for the investigation form. That’s why it takes longer time over there (secondary triage).” (Professional)*

*“The secondary (triage) is great, ok. But it should have the specific room for NCD consultation so that patient can meet our NCD staff and directly consult the patient what action should be taken next, and why. Staffs (also) need to have enough time to explain. . .if not, patient does not even know what the purpose is.”(Paramedic)*

**Figure 3.**
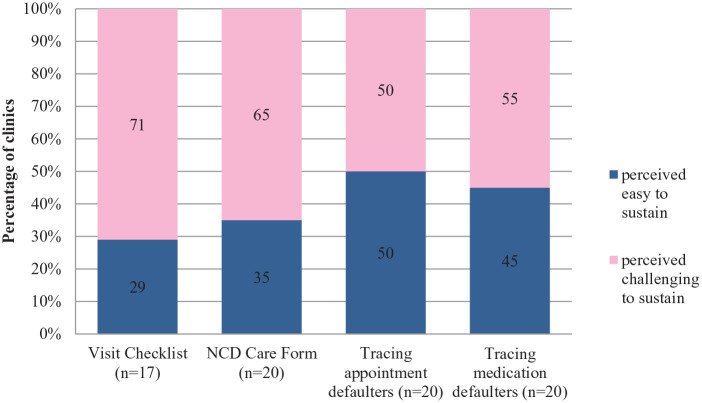
Perceived sustainability of care coordinator role.

Care Coordinator is new role introduced under EnPHC, and the main purpose is to coordinate NCD-related activities such as tracking and monitoring patient’s progress, compliance to appointment, medication, organize community education, outreach activities, and to ensure overall effectiveness of clinic management. Under EnPHC, comprehensive tracking and monitoring of patients progress was systematically standardized by using specific documentations such as NCD Care Form, and visit checklist which is put under the purview of Care Coordinator. These documentations were hoped to facilitate and equip the clinics with better tools to trace their appointment defaulters, whether it’s for routine clinic appointments, medication refills or for referrals.^15^

Quantitative assessment for care coordinator role showed for visit checklist, only 17 clinics gave feedback, whereby majority, (71%) stated it is challenging to sustain, for NCD care form, majority (65%) from the 20 clinic responded it is challenging to sustain, whereas for tracing of appointment and medication defaulters, half of them (50%) stated both as being challenging to sustain.

The qualitative feedback reflected HCP’S concerns on challenges associated with implementation of visit checklists that is; high dependency towards temporary staffs who are employed on contract basis (Temporary Contract Officer/*Pegawai Sambilan Harian* [PSH]) as these contract staffs facilitates in updating the visit checklist, shortage of manpower, limited training and facilities in ICT (computers, internet) and inconsistent and ever changing visit checklist version that caused unfamiliarity and confusion.

*“If you want to create the visit checklist, it is better to have PSH who are just designated to do the task.” (Paramedic)*

*“It’s better to have a separate internet line that is used just for the purpose of EnPHC.” (Paramedic)*

*“visit checklist. . . the version keep changing.” (Paramedic)*

For NCD Care Form, most of HCP’s expressed their views that the format of the form is not user friendly whereby patient’s details need to be written repeatedly and the form being easily misplaced.

*“Maybe it’s better to create it in a book, because for every patient, we need to write in the same details in the form. Even though it’s just about writing patient’s name, identification number etc. . . however if every day we need to write it. . .it can be tiresome too.” (Paramedic)*

For the job scope on tracing of appointment and medication defaulters, through the qualitative exploration, several issues that affects this tracing mechanism were mentioned by HCPs such as incomplete or outdated patient’s contact information, logistic issue, rigid definition for defaulter resulting in high number of patients being labeled defaulters whereby in some cases, it’s just patients delayed attendance to appointment and in certain cases patients become more dependent on staff in remembering their appointment. Some of the suggested measures that were shared by HCPs to address these limitations are extending the work contract of the contract officers (PSH) to manage the data system, NCD care form to be printed in book format, to call patients earlier or message them to remind about their follow up appointment to avoid cases of patients defaulting.

*“My plan for now is to try reducing the defaulters. . . maybe we will call patient before their TCA, then only can reduce defaulters.” (Professionals)*

*“If we have facilities to help contact the patient, it’s easy. Example WhatsApp or SMS patient. If there’s one special gadget for pharmacy defaulters, it will be easier, don’t need to call, just message the patient.” (Professional)*

**Figure 4.**
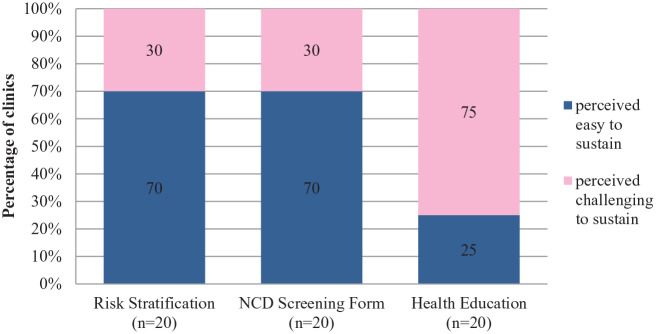
Perceived sustainability of risk stratification, NCD screening form and health education.

In NCD Screening and Risk Stratification Management, there are 3 elements; risk stratification, NCD screening form and health education and all these are implemented at Secondary Triage. The NCD screening form’s main purpose is to conduct early NCD screening to general population and to identify undiagnosed NCD issues, identify the risks and ensure these patients are referred for care continuity. Framingham Risk Score assessment was integrated as a routine screening and risk stratification practice in the clinics, whereby each patient will undergo a reassessment on a six-monthly basis. Personalized health education activities are integrated as a part of activity during routine assessments for NCD patients and it is to be provided by all paramedics. Patients are advised accordingly by paramedics based on their vital sign and risk assessment Under EnPHC, paramedics are given more prominent role and responsibilities in patient management and care whereby they were expected to manage patients that have been stratified to be of low and medium risk, leaving the high-risk cases to get acquire more attention from the medical officers.^15^

In quantitative assessment, risk stratification has been perceived to be more sustainable and only 30% stated it as challenging to sustain. During the qualitative evaluation, HCPs shared issues affecting it; limited access to calculate risk stratification as not all staffs could log in and longer time needed to calculate Framingham Score, patient’s misperception towards HCPs whereby patients misinterpreted them as using phone and not focused in work when actually HCPs are using phone for risk stratification. HCPs cited that one of the reason that contributes to this misunderstanding is due to placement of secondary triage at unsuitable location (eg, in front of patient’s waiting area).

*“(Using mobile phone to calculate) in front of patients, seems not appropriate, worried patient may comment that we are playing phone, but we are actually doing the calculations. . . .” (Paramedics)*

*“Time is not enough when seeing patient, calculating their Estimated Glomerular Filtration Rate (EGFR)*,

In quantitative assessment, 70% of the clinics reported that NCD screening form is sustainable, and through qualitative evaluation, HCPs shared concerns related to this component such as patients not being able to understand some questions in the form and refused to answer and limited supply of the forms to each clinic.

Quantitative feedback showed the component of health education was perceived to be highly challenging to be sustained by many clinics (75%). Through the qualitative assessment, many HCPs stated time constraints as the major factor affecting this component. The workflow at Secondary Triage that includes patient assessment, completion of NCD Care form and risk stratification takes time and to top it with health education activity causes overall longer clinic waiting time and more patients being impatient especially during the initial phase of the implementation. Paramedics shared as well that by conducting health education activities in haste and in unconducive environment is not helpful either for patients. Some of the suggestions by HCPs to ensure sustainability of this intervention component were sharing or rearrangement of tasks; such as stratification to be done by Medical Officers instead of paramedics, additions to staffing and space in clinics.

*“Time is needed to explain all this (health education), if not, there is no point doing it, if patients don’t understand. . .” (Professionals)*

Integrated Specialized Services (ISS) was introduced to cater comprehensive allied health services (eg, occupational therapist, nutritionist, physiotherapist, and dietician) delivered to patients if it is required. Through ISS, the inter referral mechanism was restructured with better coordination and communication platform between the facility and the allied health services. Whereas for intra referral, LOs in both clinics and hospitals were appointed to coordinate patient referrals, referral appointment date management and two-way feedback between the facilities. A referral registry was also established by EnPHC as a tracking mechanism to minimize the risk for referral defaulters. The NCD Care Form was proposed to be used as the main referral tool.^15^

**Figure 5.**
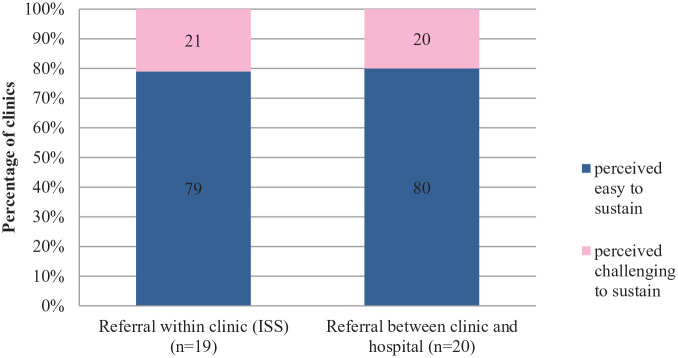
Perceived sustainability of ISS.

Based on the quantitative response received, majority of clinics perceived it’s easy to sustain this component of intervention, 79% for referral within clinic (ISS) and 80% for referral between clinic and hospital. However, during the qualitative evaluation, HCP’s shared some challenges that needed to be addressed to ensure sustainability of these structured and enhanced referral mechanism; lack of communication with referral center as well as limited or no supply of referral registry book for clinic and lack of space to conduct ISS in the clinic itself. Apart from that, another issue cited was irregular visiting schedule of ISS provider due to manpower shortage whereby only few officers are available and has to handle many clinics in certain times, sometimes it causes rescheduling of appointment dates which affects patient’s logistic convenience.

*“Nutritionist visits are scheduled once a month. In fact he comes twice in a month. . . they has the most patient, to the extend he don’t have any more available dates for appointments, we just take in patients name, only when there are available dates, then we call them. . . .” (Paramedics)*

**Figure 6.**
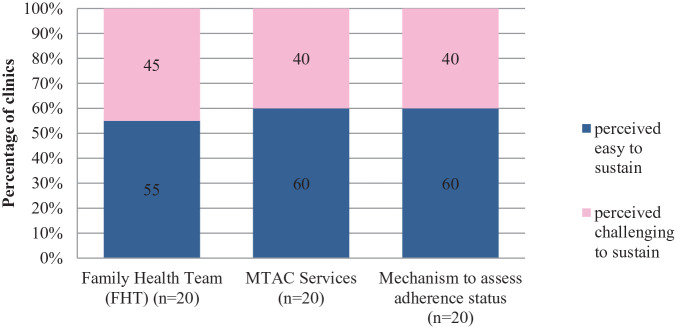
Perceived sustainability of FHT and pharmacy services.

FHT was another important component that was proposed in EnPHC and the main purpose was to upgrade the Family Doctor Concept (FDC) initiative that was implemented in stages nationwide. FDC’s previous idea was assigning a specific group of medical officers to a specific community/zone within the clinic’s catchment area. FHT adopts the similar practice but it was extended beyond medical officers whereby patients get to be seen by same group of medical teams consisting of Specialist/Medical Officers, Nurses, Medical Assistant, Pharmacists etc. The main purpose for initiation of FHT was to deliver a comprehensive medical service that comes with continuity and patient-centered approach. Familiarity between patients and clinic staffs is believed to aid in building trust, rapport and foster closer patient-doctor relationship; that leads to better health outcomes. Pharmacist’s role was also strengthened under EnPHC, through expansion of Medication Therapy Adherence Clinic (MTAC) services from diabetic-specific to the cardiovascular care as well assessment of patients’ medication adherence. Pharmacist’s work together as well with Care Coordinator to coordinate the tracing medication refill defaulter.^16^

Response from the quantitative assessment form shows that slightly more than half of the clinic managers (55%) perceived FHT as sustainable, whereas for the pharmacy services, for both MTAC services and tracking of medical adherence, 60% perceived it as sustainable. Findings from the qualitative assessment reflected that the challenges that were cited during the implementation of this initiatives were similar with other intervention components too, lack of manpower, limited space to conduct medical officers and pharmacy counseling session, language and communication barrier with patients, time constraints for both pharmacists and patients to continue with the counseling session and difficulty in tracking patients medical adherence because in certain cases medication are collected by caretakers.

*“Language barrier is also a problem. We can use an interpreter, but we don’t know whether the interpreter can interpret it as what we want to convey. Because among the staff, we can trust since we are in the same field, but if we use an outsider to translate this thing, (I am). . .afraid that he would understand and translate it differently” (Professional)*

*“MTAC is a counselling that is referred by Doctors. . . However we couldn’t provide the best due to professional constraints, infrastructure constraints. . . By right it needs certain requirements. . . if need to give counselling, we need a proper counselling room lah. . because we have a system.” (Professional)*

In overall, in both quantitative assessment and qualitative exploration among HCP’S shows acceptance and positive outlook towards the sustainability and continuous implementation of EnPHC, which was in line with the expectations of program planners and coordinators at ministry level. Even though many challenges were cited by the HCP’s in relation to the implementation and how it may affect the sustainability, but in general perspective they felt EnPHC is more systematic, aids in better management of patients and deliver care based on patient centered approach. In overall, the findings from qualitative assessment reflects HCPs as being agreeable that EnPHC intervention is more organized in providing NCD care and will benefit patients in long term. Therefore they stressed that it would be good for stakeholders to support in addressing the constraints and limitations faced for a successful outcome and sustainability of the intervention in a long term.

*“I think in terms of process of care, it’s better because systematic, It’s just the (work) load and facility wise la,more space needed for patient’s comfort “ (Professional)*

## Discussions

HCP’s in this evaluation study were generally satisfied with the implementation and foresee that the initiatives aided in improving the quality of health service especially in terms of patient’s disease management. Based on the feedbacks shared by HCP’s, the intervention projected overall improvement in patients’ continuity of care, however this evaluation does not include assessment of changes in biochemical or other clinical markers of disease in those with diabetes and hypertension. Reviews from other similar studies often discussed increased community awareness and improved clinical processes as important markers of intervention but to observe modest improvements in clinical markers, it may take up to 3 years for changes to be apparent after an intervention.^17^

HCP’s issues and challenges related to the sustainability of each intervention component were discussed in this paper, mainly involving lack of resources in terms of manpower and personnel, limits in the availability of clinic space, lack of ICT facilities, unreliable online system, etc. In addition to resource constraints, HCP’s also shared some elements of intervention criteria that need to be re-examined and redefined, such as the placement of more appropriate primary triage workers, a more refined description of defaulters since, in certain instances, their appointments are only cases of patient delayed attendance.

Health systems that are sustainable are the ones that have sufficient resources to meet their objectives and able to adapt with environment changes, able to manage challenges and updated with developments.^16^ Relating this with the findings from this study, lack of resources in terms of manpower and available facilities (clinic structure, space, ICT) emerged as main challenges faced during the implementation of most of the intervention component. HCPs foresee the difficulties in sustaining the interventions if these issues are not resolved. Lack of staff continuity was stated as a common threat to sustainability whereas staff continuity was seen as a facilitator of sustainability. Many past studies in health care settings; particularly those in clinics or hospitals that assessed barriers and facilitators to sustainability highlighted funding, organizational factors like support from champions, continuous supervisions, and practitioner/workforce characteristics as highly influential for sustainment of any health intervention initiatives.^18^ Limitations in funding and workforce, lack monitoring and evaluations to track and improve performance, as well as weak political commitment has caused many public health programs to fail. A study on assessment of health professions educators whom are engaged in implementing education innovations, identified the importance of stakeholder support and adequate resources as one of the key influencing factors that impact the sustenance of any innovations.^19^ Many other research reviews on sustainability had highlighted as well the importance of infrastructure and stability of the work force as key component influencing sustainability.^20^

In this study, some clinic managers shared how they adapted some intervention requirements based on their clinical setting, such as placement of health attendants rather than medical assistants at primary triage to save on manpower during busy clinic days, and in another clinic, clinic managers made some changes in clinical role such as risk stratification to be prepared by doctor compared to earlier by nurses at secondary triage reduce patients waiting time and queue. Relating to this, it can be seen that clinical managers and LOs in EnPHC facilities plays important role in leading, continuously evaluating the implementation process, being innovative by adapting and apply necessary changes if needed to ensure smooth running of the project. Presence of a key person is essential to facilitate innovative and sustainable practices whereby the intervention may be stabilized by the positive influence of this member, establishing networking and shared responsibilities among other members in the team.^21^ This can be identified in similar study by Wiltsey Stirman et al^22^ that stated sustainability of intervention are influenced by several key factors; innovation characteristics (eg, fit and effectiveness of the intervention), context (eg, culture and leadership), capacity (eg, funding and resources), and processes and interactions (eg, shared decision-making and adaptation/alignment). In another study, one of the needs identified for sustainability was capacity and motivation of work agents to adapt to innovation, compatible with new and existing roles and tasks and this often relates to workers at the front line as the main group that needs to be flexible toward the adaptation^19^ and engagement of stakeholders also seen as another crucial and frequently associated factor for sustainability especially in public health initiatives.^22^ Findings of this study also showed how clinic managers and LOs improve the implementation process with their tried and tested work procedures and made adaptations accordingly, thus reflecting the component of constant evaluation conducted by the managers to upgrade their work performances, something Gruen et al^23^ stated as a significant element that influences sustainability; ongoing cycles of reflection, planning, and action.

Overall findings of the study reflected on how the 4 key objectives of the EnPHC intervention was achieved despite difficulties and challenges associated with implementation and sustaining the intervention components. HCPs shared their experience after the implementation of EnPHC such as systematic appointment recoding and filing system, better management and care delivered to patients, and stakeholders especially financial funders being more sensitive to the needs of the clinic.^15^ These responses are parallel with few studies that enlightens on factors associated with sustainability; providers’ commitment to a best practice service delivery model, worker acceptability of the intervention^16^ and organizational capacity and ability to adapt to a changing environment.^23^ The study findings also reflected the commitment and initiative characteristics that were presence among the clinic managers and HCPs involved in the intervention clinic facilities. Apart from this, adequate resources and relationships^21^ are noted too as important factors in enhancing sustainability therefore it important for policy/decision-makers and healthcare managers to have a continuous communication on needed resources and change strategies that can improve interventions that are introduced.^14^

## Limitations

One of the limitations identified for this study is the nature of the quantitative data analysis which were purely descriptive and involved simple counts and percentages. This is due to the number of facilities involved in evaluation which was limited to only 20 clinic and each participating clinic were considered as one unit. For the qualitative analysis, the exploration on sustainability were discussed along with their quantitative feedback, thus limiting the perceptions of HCPs, whereby most of their responses were confined to issues and challenges in implementing the intervention and how they foresee it as an hindrance for sustainment of the intervention in long term whereas not much was reflected on positive aspects such as the driving force or motivating factors within themselves or the organization that had made the intervention implemented regardless of all the lacking and how this positive aspect could aid in sustainment of the intervention in future.

## Conclusion

This study has identified perceived sustainability and challenges associated with each intervention components under EnPHC. It’s crucial to address the issues highlighted by HCPs mainly in inadequate resources (manpower, infrastructure facilities, IT facility, etc.) and stabilize overall management of the intervention to ensure the smooth workflow of this intervention. Based on suggestions from HCP’s on aspects that can be improved in this interventions, it is important for decision makers, managers, and implementers at all level; ministry, state/district health offices, and primary health care facilities to strategize and plan systematically in improving the intervention components based on feedback received, and expand the resources needed to ensure sustainability and overall successfulness of this program in long term.

## Supplemental Material

sj-pdf-1-jpc-10.1177_21501327211014096 – Supplemental material for Enhanced Primary Health Care Intervention: Perceived Sustainability and Challenges Among Implementers Click here for additional data file. Supplemental material, sj-pdf-1-jpc-10.1177_21501327211014096 for Enhanced Primary Health Care Intervention: Perceived Sustainability and Challenges Among Implementers by Komathi Perialathan, Mohammad Zabri Johari, Norrafizah Jaafar, Kong Yuke Lin, Low Lee Lan, Nur Aliyah Sodri and Siti Nur Nabilah Mohd Yunus in Journal of Primary Care & Community Health
