# Supplementary material for: Enhanced Primary Health Care Intervention: Perceived Sustainability and Challenges Among Implementers
Source: J Prim Care Community Health. 2021 May 8;12:21501327211014096. doi: 10.1177/21501327211014096 (PMC8114286; doi:10.1177/21501327211014096)
Supplement: sj-pdf-1-jpc-10.1177_21501327211014096 – Supplemental material for Enhanced Primary Health Care Intervention: Perceived Sustainability and Challenges Among Implementers [file sj-pdf-1-jpc-10.1177_21501327211014096.pdf]

SENARAI SEMAK TAHAP PELAKSANAAN INTERVENSI *ENHANCED PRIMARY HEALTH CARE*
Kajian EnPHC Process Evaluation (Fasa 3)

Arahan:

1. Borang ini perlu **dilengkapkan** oleh ***Liaison Officer*** **(LO)** di Klinik Kesihatan yang **menjalankan intervensi** *Enhanced Primary Health Care* (EnPHC) di negeri Selangor dan Johor.
2. Maklumat yang diperlukan adalah berdasarkan pengalaman melaksanakan intervensi EnPHC dari tempoh Oktober 2017 sehingga Mac 2018.
3. Borang yang dilengkap perlu dikembalikan sebelum atau pada 31 Mac 2018 secara muat naik ke e-mel berikut: enphc.psh@moh.gov.my; atau secara fax ke no berikut: +603 3359 6014; atau secara pos ke alamat berikut:

   Pasukan Penyelidik Enchanced Primary Healthcare
   c/o: Dr. Low Lee Lan (Penyelidik Utama)
   Institut Penyelidikan Sistem Kesihatan
   Suites 55-1, 55-2, 55-3, 55-4. Setia Avenue,
   No.2 Jalan Setia Prima S U13/S. Seksyen U13 Setia Alam.
   40170 Shah Alam Selangor.

TERIMA KASIH ATAS BANTUAN ANDA DALAM MENJAYAKAN KAJIAN INI.

Klinik Kesihatan :______________________________

Tarikh diisi :______________________________

Liaison Officer (LO) :______________________________

SENARAI SEMAK TAHAP PELAKSANAAN INTERVENSI *ENHANCED PRIMARY HEALTH CARE*
Kajian EnPHC Process Evaluation (Fasa 3)

1. Adakah terdapat **PERUBAHAN** dalam **ARAHAN PERLAKSANAAN** (contoh: BPKK, LO Negeri, Pasukan Pemantauan, Mentor Klinik, Pengarah Negeri, dll) bagi komponen intervensi *Enhanced Primary Health Care*. Jika ada, sila isi maklumat dalam kotak yang berkaitan.

**Intervensi**	**Terdapat perubahan pada bulan & tahun:**	**Arahan daripada:**	**Maklumat Perubahan:**
Contoh 1: Audit checklist	November 2017	BPKK	Penambahbaikan kriteria audit
Contoh 2: Primary Triage Counter	Januari 2018	LO Negeri;<br>Pasukan Pemantauan	Kedudukan meja dari dalam ruang menunggu ke pintu masuk utama
Primary Triage Counter			
Secondary Triage Counter			
Audit Criteria Document			
Care Coordinator – appointment defaulters tracing			
Care Coordinator – medicine pharmacy defaulters			

SENARAI SEMAK TAHAP PELAKSANAAN INTERVENSI *ENHANCED PRIMARY HEALTH CARE*
Kajian EnPHC Process Evaluation (Fasa 3)

Referral Mechanism			
Population Registry			
Visit Checklist			
Lain-lain arahan perubahan:			

**Sekirannya tiada arahan perubahan dalam tempoh tersebut, sila tulis TIADA.*

2. Sila beri pandangan anda terhadap **KEUPAYAAN KLINIK** ini untuk meneruskan **(*sustainability*)** intervensi *Enhanced Primary Health Care* untuk **JANGKAMASA PANJANG**. Sila beri jawapan berdasarkan skala berikut (**1**= amat sukar, **2**= sukar, **3**= mudah, **4** = amat mudah).

No.	Intervensi	Skala (1 – 4)	Jika pilihan skala 1 atau 2, kenapa?
	Contoh:<br>(i) Borang NCD care form<br>(ii) Visit checklist<br>(iii) Mechanism to trace pharmacy medicine defaulters	 2<br>1<br>1	 Borang yang dibekalkan tidak mencukupi.<br>Lantikan PSH hanya sementara.<br>Masalah internet
1.	Population Registry (MOVEs)		
2.	Primary Triage Counter		
3.	Secondary Triage Counter		
4.	Health Education Services at Secondary Triage		
5.	Risk Stratification Criteria (Framingham Risk Score)		
6.	NCD Screening Form		
7.	Audit enhanced PHC - Customer Satisfaction Survey		
8.	Audit enhanced PHC – appointment defaulter		
9.	Audit enhanced PHC – target patients Risk stratification		
10.	Audit enhanced PHC - NCD care process		
11.	MTAC Services		
12.	Mechanism to assess adherence status		
13.	Integrated Specialised Services (ISS)		
14.	Family Health Team (FHT)		
15.	Visit Checklist		
16.	NCD Care Form		
17.	Mechanism to trace appointment defaulters		
18.	Mechanism to trace pharmacy medicine defaulters		
19.	Referral Registry		

*Sekiranya terdapat maklumat tambahan, sila kepilkan dalam lampiran berasingan.

3. Sila nyatakan dengan terperinci sekiranya terdapat isu dalam pelaksanaan intervensi *Enhanced Primary Healthcare* sehingga kini.

**Intervensi**	**Isu Pelaksanaan**
Contoh: NCD Care Form	1. Borang besar sangat, jadi tebal bila disimpan dalam fail/buku pesakit<br>2. Borang perlu ditulis setiap kali pesakit datang untuk temujanji; menyusahkan.<br>3.
Primary Triage	1.<br>2.<br>3.
Secondary Triage	1.<br>2.<br>3.
Audit EnPHC	1.<br>2.<br>3.
Care Coordinator – appointment defaulters tracing	1.<br>2.<br>3.
Care Coordinator – medication defaulters tracing	1.<br>2.<br>3.
Referral Mechanism	1.<br>2.<br>3.
Visit Checklist	1.<br>2.<br>3.

*Sekiranya terdapat maklumat tambahan, sila kepilkan dalam lampiran berasingan.
